# Structural Rearrangement in Cyclic Cu(II) Pyridyltriazole Complexes: Oxidation of Dabco to Oxalate and CO_2_ Conversion to Carbonate

**DOI:** 10.3390/molecules30071430

**Published:** 2025-03-24

**Authors:** Uttam R. Pokharel, Frank R. Fronczek, Andrew W. Maverick

**Affiliations:** 1 Department of Physical & Applied Science, University of Houston—Clear Lake, Houston, TX 77058, USA; 2 Department of Chemistry, Louisiana State University, Baton Rouge, LA 70803, USA; ffroncz@lsu.edu

**Keywords:** rearrangements, metal–organic supramolecules, dabco, carbonate and oxalate bridges, copper(II) complexes, pyridyltriazole, oxidative transformation, host–guest complex

## Abstract

Structural rearrangements in metal–organic supramolecules constructed from the coordination of Cu(II) with *m*-xpt (*m*-xylylenebis(pyridyltriazole)) are investigated upon their interaction with 1,4-diazabicyclo[2.2.2]octane (dabco) and carbon dioxide-enriched air. The binuclear [Cu_2_(*m*-xpt)_2_]^4+^ complexes react with dabco to produce a carbonate-bridged trinuclear complex, [Cu_3_(*m*-xpt)_3_(µ-CO_3_)]^4+^, and an oxalate-bridged binuclear complex, [Cu_2_(*m*-xpt)_2_(µ-C_2_O_4_)]^2+^, where carbonate and oxalate likely originate from CO_2_ and dabco, respectively. The trinuclear complex reassembles the original dimer upon the removal of the carbonate ion. Similarly, polymeric [Cu(*o*-xpt)(PF_6_)]_n_, formed from Cu(I) and *o*-xpt (*o*-xylylenebis(pyridyltriazole)) coordination, undergoes oxidation in CO_2_-enriched air to yield a tetranuclear Cu(II) complex, Cu_4_(*o*-xpt)_3_(μ_4_-CO_3_)(μ_2_-OH)(μ_2_-OCOCH_3_)^4+^. The reaction progress is monitored by UV-Vis spectroscopy, and the major products are characterized by single-crystal X-ray diffraction.

## 1. Introduction

The design of coordination-driven metal–organic cages has emerged as an important subfield of supramolecular chemistry recently due to their diverse potential applications in areas such as catalysis [1,2,3,4,5,6], separation [7,8,9,10,11], and sensing [12,13,14,15]. The rational design of these supramolecular cages relies on the careful consideration of the stereochemical preference of the transition metal ions, the coordination sites, and the geometrical preference of multidentate ligands, as well as the solubility profile of counter-anions [16,17,18]. These cages possess well-defined cavities capable of encapsulating guest molecules through secondary interactions, including Lewis acid–base interactions [19], π-π stacking [20,21], and hydrogen bonding [22].

Typically, the interactions of guest species preserve the structural topology of the host molecules in supramolecular host–guest interactions. However, in some cases, guest species can induce the structural rearrangement of the host [23,24]. Zonta and co-workers demonstrated that diacids as guests can trigger the disassembly of macrocyclic cages formed from tris(2-pyridylmethyl)amine and a zinc(II) complex via imine condensation [25,26]. Despite these findings, the role of guest species in simultaneously inducing disassembly and mediating subsequent transformations or reassembly remains largely underexplored.

Dabco (1,4-diazabicyclo[2.2.2]octane) is a bicyclic compound containing two nitrogen atoms in a rigid, cage-like structure. It is widely used as a base or nucleophile in organic synthesis, catalysis, and materials science [27]. In coordination chemistry, dabco often serves as a guest molecule in metal–organic self-assemblies due to its bidentate coordination capability and basicity [19,28,29,30]. While it has been employed as a structural template in the assembly of metal–organic complexes [31,32], there are limited reports of its chemical transformation during the metal–organic self-assembly process. Notably, Knope and Cahill observed that dabco undergoes oxidation to oxalate when heated with uranium oxynitrate hexahydrate and tetraethylammonium hydroxide under hydrothermal conditions [33]. This suggests that dabco can undergo chemical transformation in specific reaction environments.

**Figure 1:**
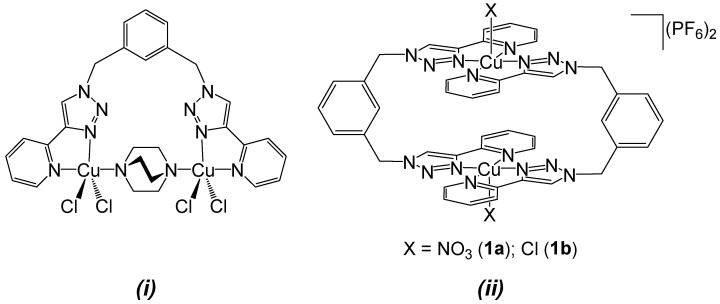
Examples of copper(II) complexes assembled from the *m*-xpt ligand. (***i***) Dabco-bridged binuclear complex with one *m*-xpt ligand; and (***ii***) metallacyclic complexes used as starting materials in this study: [Cu_2_(*m*-xpt)_2_(NO_3_)_2_](PF_6_)_2_ (**1a**) and [Cu_2_(*m*-xpt)_2_Cl_2_](PF_6_)_2_ (**1b**).

Previously, we synthesized Cu(II) complexes of tetradentate pyridyltriazole-based ligands separated by a 2,7-dimethylnaphthalene spacer [19]. In this macrocyclic complex, the Cu···Cu separation was 7.5385(9) Å when the dabco occupied its cavity as a guest. Similarly, the treatment of *meta*-xylylenebis(pyridyltriazole) (*m*-xpt) with CuCl_2_ in the presence of dabco yielded a binuclear complex [Cu_2_(*m*-xpt)(dabco)]^4+^, which exhibited a Cu···Cu separation of 6.634(1) Å (Figure 1*i*). These findings prompted us to investigate the possibility of incorporating dabco as an internal guest of dimeric macrocycles constructed from *m*-xpt and Cu^2+^. Herein, we present the structural rearrangements caused by dabco in these macrocycles (Figure 1*ii*) and the concurrent formation of oxalate and carbonate species during the reassembly process. As a complement to this study, we report the reactivity of Cu(I) complexes of *ortho*-xylylenebis(pyridyltriazole) (*o*-xpt) for the activation of small guest molecules.

## 2. Results and Discussion

### 2.1. Interactions of Supramolecular Complexes with Dabco in Air

**Figure 2:**
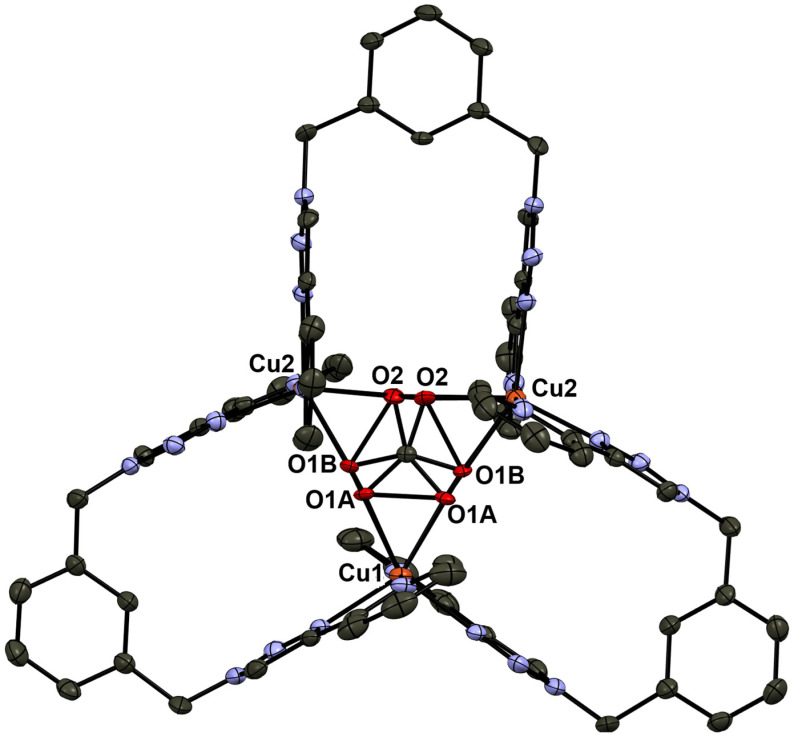
Ellipsoid plot of [Cu_3_(*m*-xpt)_3_(μ-CO_3_)](PF_6_)_4_, ([**3**](PF_6_)_4_). The complex has crystallographically imposed two-fold rotation symmetry, with the axis approximately vertical in this drawing. This results in the disorder of the carbonate anion. In this and other crystal structure illustrations, ellipsoids are at the 50% probability level, and hydrogen atoms, solvents, and counterions are omitted for clarity.

**Figure 3:**
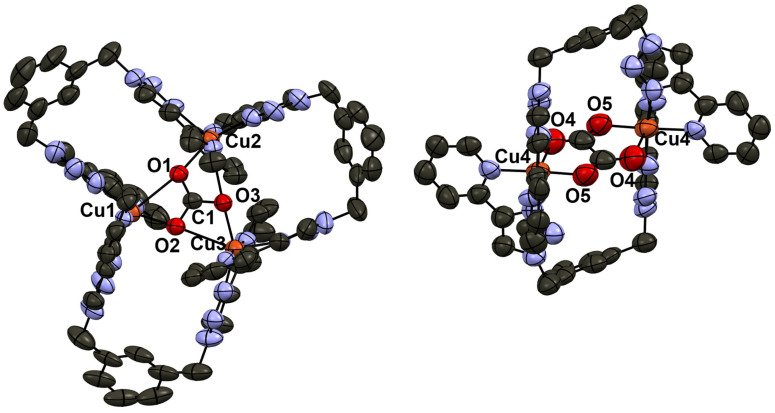
Ellipsoid plot of the two cations in [Cu_3_(*m*-xpt)_3_(μ-CO_3_)]_2_[Cu_2_(*m*-xpt)_2_(μ-C_2_O_4_)](PF_6_)_10_, [**3**]_2_[**4**](PF_6_)_10_.

Figure 1 shows the two Cu(II) complexes employed as starting materials in our experiments: [Cu_2_(*m*-xpt)_2_(NO_3_)_2_](PF_6_)_2_ (**1a**) and [Cu_2_(*m*-xpt)_2_Cl_2_](PF_6_)_2_ (**1b**). These [Cu_2_(*m*-xpt)_2_]^4+^ complexes display of range of Cu···Cu distances in the solid state, some of which are 7.6 Å or greater [19]. This is more than enough to accommodate dabco as an internal guest molecule. Our initial investigation of host–guest interactions between these macrocycles with dabco in air showed a subtle color change in complex **1a** in acetonitrile and a pronounced color change in complex **1b** in DMF upon the addition of five equivalents of dabco to each complex (Figure S1). The slow vapor diffusion of diethyl ether facilitated crystallization, yielding blue plate-shaped crystals at the upper part of the test tube. X-ray crystallographic analysis confirmed these as a carbonate-bridged trinuclear complex, [Cu_3_(*m*-xpt)_3_(µ-CO_3_)](PF_6_)_4_, [**3**](PF_6_)_4_ (Figure 2). Meanwhile, the needle-shaped green crystals that formed at the lower part of the test tube were identified as the carbonate-bridged trinuclear complex ([**3**]^4+^) co-crystallized with an oxalate-bridged binuclear complex, [Cu_2_(*m*-xpt)_2_(µ-C_2_O_4_)]^2+^ ([**4**]^2+^), with a stoichiometry of [**3**]_2_[**4**](PF_6_)_10_ (Figure 3).

In the structure of [**3**](PF_6_)_4_ (Figure 2), the cation lies on a two-fold axis (passing through one *m*-xylylene group, the central carbonate C, and Cu1) in the crystal. The carbonate anion is bound asymmetrically; Cu1···Cu2 4.6058(8) and Cu2···Cu2′ 4.6126(8) Å. The structure of [**3**]_2_[**4**](PF_6_)_10_ (Figure 3) contains two different cations. The carbonate-bridged trimer [**3**]^4+^ lies in a general position, with Cu···Cu distances of 4.534(2), 4.705(1), and 4.583(1) Å. The oxalate-bridged dimer [**4**]^2+^ lies on an inversion center; Cu4···Cu4′ 5.501(2) Å.

### 2.2. Spectroscopic Monitoring of the Interaction Between Supramolecules and Dabco

**Figure 4:**
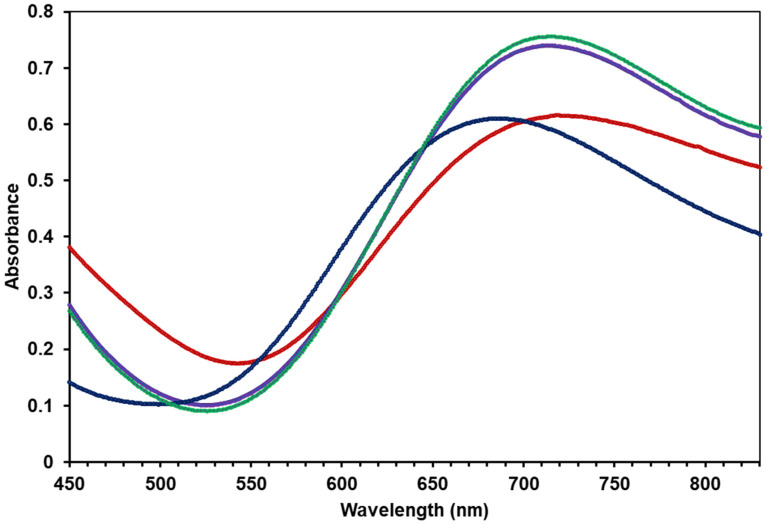
A portion of the electronic spectrum of a 3.75 mM solution of [Cu_2_(*m*-xpt)_2_(NO_3_)_2_](PF_6_)_2_ (**1a**) in acetonitrile (▬▬), with one equivalent of dabco (▬▬) under N_2_. The solution was then kept under CO_2_ for 10 min (▬▬) and for 24 h (▬▬).

These observations prompted us to investigate the reactions under controlled conditions and monitor the progress by UV-Vis spectrometry. Upon adding one equivalent of dabco under a nitrogen atmosphere, the primary d-d absorption band of compound **1a** in CH_3_CN showed a red shift from 686 nm to 718 nm. No further shift in absorbance was observed with additional dabco, suggesting the formation of a 1:1 host–guest complex. Microanalysis of the green microcrystalline product (**2**) supported the formation of the 1:1 adduct with dabco (see experimental section). The compound may have contained dabco internally bound within the complex or externally coordinated, potentially forming a chain-like arrangement: –Cu_2_–dabco–Cu_2_–dabco–. Unfortunately, we were unable to obtain suitable crystals for X-ray analysis. The UV-vis absorption spectrum of compound **2** showed a minimal shift (ca. 5 nm) in the presence of CO_2_, but its intensity was increased (Figure 4). The spectrum of this solution was very similar to that of [**3**](PF_6_)_4_ in acetonitrile (λ_max_ 715 nm), suggesting that **2** was converted to the carbonate-bridged trinuclear complex.

The above changes primarily reflected the host–guest binding of **1a** with dabco. In contrast, the treatment of **1b** with dabco led to reduction to Cu(I). This difference in behavior is shown in Figure S1. Attempts to monitor the reaction of complex **1b** with dabco in acetonitrile were unsuccessful due to its low solubility. However, UV-Vis monitoring of **1b** in DMF under nitrogen showed a gradual increase in signal intensity at ~440 nm (Figure S2), which we attribute to a metal-to-ligand charge transfer (MLCT) band, characteristic of Cu(I) complexes.

### 2.3. Role of Water in Carbonate/Oxalate Formation

Repeated experiments revealed that complex **1a** produces more carbonate-bridged complex [**3**]^4+^ in bulk solvents than in dry solvents. This suggests that the carbonate ions in [**3**]^4+^ are generated in situ by the hydration of carbon dioxide in the presence of dabco as a base. Dabco reacts with residual water in the solvent and carbon dioxide to form bicarbonate. The bicarbonate coordinates with Cu(II) and then loses a proton to produce carbonate. In fact, one of the byproducts of the reaction is protonated dabco molecules linked by intermolecular hydrogen bonding [34] (Figure S3). Complex **1b** is more effective for forming the co-crystallized oxalate/carbonate complex [**3**]_2_[**4**](PF_6_)_10_ than **1a**. Based on the yields of [**3**](PF_6_)_4_ and [**3**]_2_[**4**](PF_6_)_10_ from **1b**, the overall amount of oxalate complex [**4**]^2+^ formed is approx. 10% that of the carbonate complex [**3**]^4+^.

### 2.4. Spectroscopic Characterization

The crystals of [**3**](PF_6_)_4_ and [**3**]_2_[**4**](PF_6_)_10_ were manually separated for further analysis. The ESI-MS analysis of the trinuclear carbonate complex [**3**](PF_6_)_4_ showed a monoisotopic peak at 1663.2112 corresponding to a fully reduced trimer [Cu_3_(*m*-xpt)_3_(PF_6_)_2_]^+^. However, we were not able to detect any peak associated with CO_3_^2−^, suggesting that the coordination of carbonate ions to the metal centers is weak. The IR of the product showed a prominent peak at 1459 cm^−1^, corresponding to the C=O stretching (Figure S4) of the carbonate group, as reported in carbonate-bridged Cu complexes [35]. The co-crystallized compound [**3**]_2_[**4**](PF_6_)_10_ exhibited signals at 1659 cm^−1^ and 1457 cm^−1^ in IR (Figure S5), corresponding to C=O stretching in the oxalate and carbonate. ESI-MS analysis showed a monoisotopic peak at 1147.1636, corresponding to [Cu_2_(*m*-xpt)_2_(C_2_O_4_)(PF_6_)]^+^.

### 2.5. Sources of Carbonate and Oxalate

Carbon dioxide, oxygen, and water are frequently present in metal–organic self-assembly reactions. The role of carbon dioxide in carbonate formation is well documented [36,37,38,39,40,41,42,43,44,45]. There have been reports of oxalate formation during self-assembly [46,47,48,49,50,51], though its sources are not always clearly identified. In our previous study, we demonstrated the high affinity of both complexes **1a** and **1b** to host oxalate. Upon the reduction of the Cu(II) centers to Cu(I) using ascorbic acid, followed by the exposure of the mixture to air, oxalate was observed as an oxidation product of ascorbic acid [52].

**Scheme 1:**
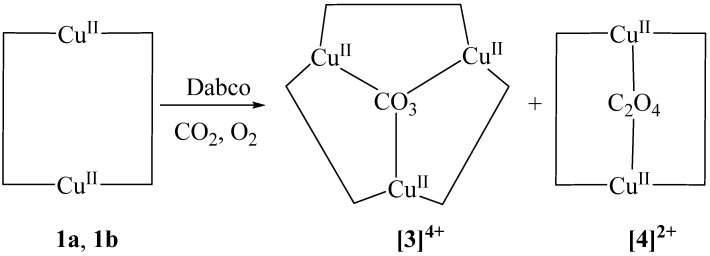
Rearrangement of macrocyclic Cu-*m*-xpt complexes.

Although the chloro group is normally a better electron donor than nitrate for stabilizing Cu(II) centers, the redox potentials of the two complexes are nearly identical (−0.27 V for complex **1a** and −0.28 V for complex **1b** versus Fc/Fc^+^). Interestingly, the dihedral angle between the two pyridyltriazole moieties around the Cu(II) center is greater for **1b** than for **1a**. This greater twisting in the coordination environment of **1b** might hinder the efficient coordination of dabco, causing dabco to act as a reducing agent rather than as a guest in this complex. We propose that the oxidation of dabco is responsible for the formation of oxalate, and that carbon dioxide is the source of carbonate. The carbonate and oxalate ions serve as templates for the self-assembly of trinuclear complex [**3**]^4+^ and binuclear complex [**4**]^2+^ (Scheme 1).

### 2.6. Release of Carbonate Ion from Complex [**3**]^4+^

The release of carbonate ions from trinuclear complex [**3**]^4+^ was observed upon treatment with BaBr_2_ in DMF. Upon the addition of BaBr_2_ to the solution, the color changed from blue to green, accompanied by the formation of a white precipitate. When treated with dilute HCl, the precipitate dissolved with the effervescence of CO_2_, confirming the formation of BaCO_3_. The slow diffusion of diethyl ether into the green solution yielded the “empty” macrocycle [Cu_2_(*m*-xpt)_2_Br_2_](PF_6_)_2_, as evidenced by preliminary crystallographic data (Figure S6). The empty macrocycle contained bromo groups occupying the axial positions with a Cu···Cu separation of 7.592 Å.

In the presence of acid, the trinuclear complex [**3**]^4+^ also rearranged into a binuclear species. The addition of dilute HCl to an acetonitrile solution of complex [**3**](PF_6_)_4_ produced a green–blue precipitate, which dissolved in DMF. The slow vapor diffusion of diethyl ether into the DMF solution gave a dark-green crystalline product. X-ray crystallographic analysis revealed the formation of [Cu_2_(*m*-xpt)_2_Cl_2_]Cl_2_, as reported previously [19].

### 2.7. Synthesis of Cu(I) Complex of ortho-Xylylenebis(Pyridyltriazole) (o-xpt) and Its Reactivity with CO_2_-Enriched Air

**Scheme 2:**
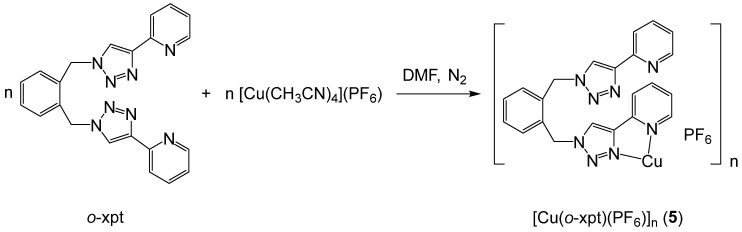
Synthesis of [Cu(*o*-xpt)(PF_6_)]_n_ (**5**).

**Figure 5:**
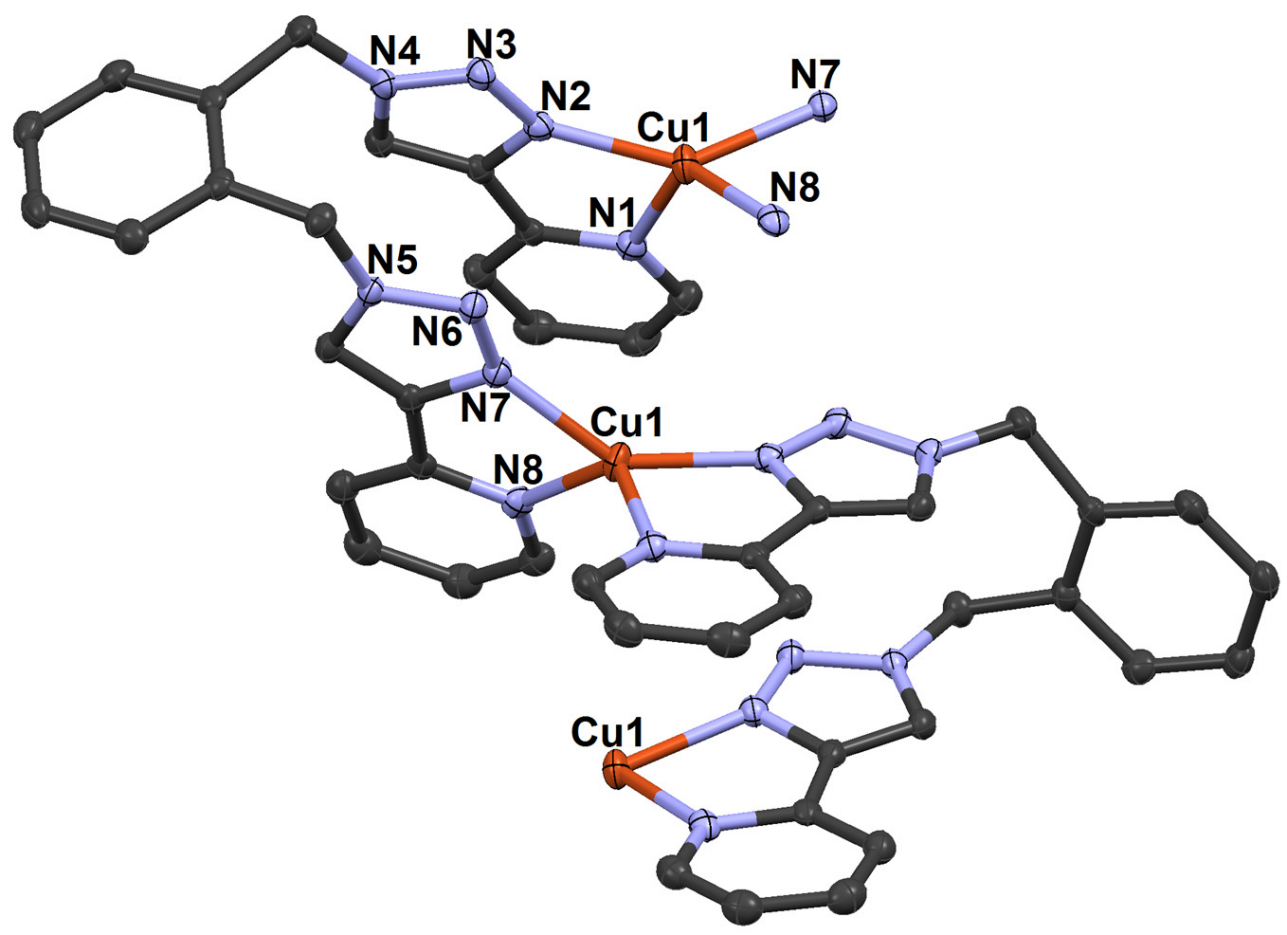
Ellipsoid plot of [Cu(*o*-xpt)(PF_6_)]_n_ (**5**). The Cu···Cu separation is 4.0304(5) Å.

In our previous work, we reported the formation of a carbonate-bridged trinuclear complex, [**3**]^4+^, when the Cu(I) complex of *m*-xpt was oxidized in CO_2_-enriched air [52]. However, we were unable to determine the structure of the Cu(I) complex by X-ray crystallography. To investigate the impact of Cu···Cu separation on CO_2_ fixation to CO_3_^2−^, we synthesized a Cu(I) complex of *ortho*-xylylenebis(pyridyltriazole) (*o*-xpt) and crystallized this under a nitrogen atmosphere. The product was a bright yellow microcrystalline solid (Figure S7), and single-crystal X-ray analysis revealed the formation of a polymeric network of [Cu(o-xpt)(PF_6_)]_n_ (**5**) (Scheme 2; Figure 5). Although the Cu···Cu separation in this complex was similar to the Ag···Ag separation in [Ag_2_(*o*-xpt)_2_]^2+^, as reported by Crowley et al. [53], the dimerization in this case was possibly less favorable due to the preferred tetrahedral geometry of Cu(I) coordination.

In the structure of **5**, the two Cu(pyridyltriazole) planes made an angle of 65.34°, representing some distortion compared to the ideal angle of 90°. This may be compared to the values found in three other [Cu^I^(pyridyltriazole)_2_]^+^ structures: 63.85° [54], 85.18° [55], and 89.75° and 81.46° [56]. It was also well within the range of the interligand angles for [Cu(2,2′-bpy)_2_]^+^ structures (37.6–85.7°, 95 examples in 55 structures) [57].

**Figure 6:**
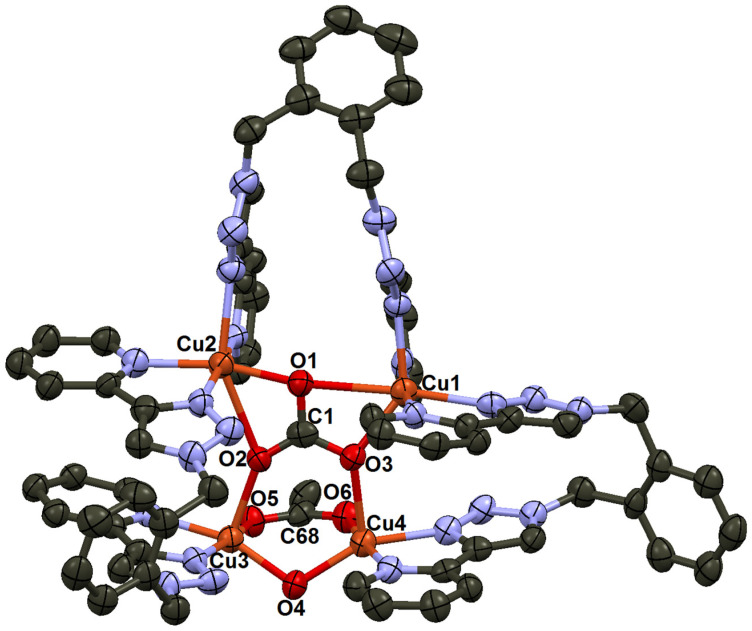
Ellipsoid plot of [Cu_4_(*o*-xpt)_3_(μ_4_-CO_3_)(μ_2_-OH)(μ_2_-OCOCH_3_](PF_6_)_4_ (**6**). One of the two independent cations in the asymmetric unit is shown. Cu1···Cu2, 4.412(2) Å; and Cu3···Cu4 3.171(2) Å.

When a DMF solution of complex **5** was exposed to CO_2_-enriched air, it gradually changed to blue. Attempts to grow crystals from DMF resulted in the formation of a non-crystalline blue precipitate. Therefore, the precipitate was redissolved in acetonitrile, and diethyl ether vapor was diffused into the solution. X-ray crystallographic analysis of the resulting product revealed the formation of a tetrameric complex, [Cu_4_(*o*-xpt)_3_(μ_4_-CO_3_)(μ_2_-OH)(μ_2_-OCOCH_3_)_4_](PF_6_)_4_ (**6**) (Figure 6). Unlike complex [**3**]^4+^, the carbonate ion in this complex bridged four Cu(II) centers, two of which were further linked by hydroxy (OH^−^) and acetate (CH_3_COO^−^) ligands. The carbonyl stretch of the acetate bridge appeared at 1668 cm^−1^, while the carbonate stretch was observed at 1458 cm^−1^ (Figure S8). The acetate was likely formed via the hydrolysis of acetonitrile, while the hydroxy bridge (μ-OH) between the Cu(II) centers may have arisen from water coordination followed by proton loss. In the crystal structure of **6**, there were two crystallographically independent [Cu_4_(*o*-xpt)_3_(μ_4_-CO_3_)(μ_2_-OH)(μ_2_-OAc)]^4+^ cations with very similar conformations. One of them is shown in Figure 6.

## 3. Materials and Methods

### 3.1. General Procedures

All commercially available reagents and solvents were purchased from Sigma-Aldrich (St. Louis, MO, USA) and Alfa Aesar (Ward Hill, MA, USA) and were used as received. NMR spectra were recorded on a Bruker 400 MHz (Avance) spectrometer (Bruker, Billerica, MA, USA). ESI mass spectra were measured on an Agilent 6230 instrument (Agilent Technologies, Santa Clara, CA, USA). FTIR spectra were recorded on a Bruker Tensor 27 spectrometer (Bruker, Billerica, MA, USA) equipped with a diamond crystal ATR sample holder in a range between 400 and 4000 cm^−1^. Elemental analyses were performed by M-H-W Laboratories, Phoenix, AZ, USA. UV-visible spectra were recorded in a custom-designed reaction flask connected to the cuvette using an Aviv 14DS spectrometer (Aviv Biomedical, Inc., Lakewood, NJ, USA). The ligands *o*-xpt [53] and *m*-xpt [19] were synthesized following the literature procedures. For the preparation, structures, and electrochemical properties of the complexes [Cu_2_(*m*-xpt)_2_X_2_](PF_6_)_2_ [where X = NO_3_ (**1a**), Cl (**1b**)] (Figure 1*ii*), see [52] and references therein.

### 3.2. Experimental Procedures

**[Cu_2_(*m*-xpt)_2_](PF_6_)_4_·dabco (2):** In a nitrogen-purged Schlenk flask, complex **1a** (100 mg, 0.08 mmol) was dissolved in dry acetonitrile (20 mL), and dabco (25 mg, 0.23 mmol) was added. The complex changed its color from blue to blue–green as soon as dabco was added. N_2_ purged diethyl ether was slowly diffused into the reaction mixture via a cannula over a period of 4–5 days; by then, the green solid was deposited as a microcrystalline product. The mother liquor was decanted, and the product was washed with N_2_-purged diethyl ether. Then, it was dried under vacuum to give **2** (87 mg) as a green solid. ESI-MS: *m*/*z* 1349.0783, [Cu_2_(*m*-xpt)_2_(PF_6_)_3_]^+^ (calcd 1349.0821). Anal. Calcd [Cu_2_(*m*-xpt)_2_](PF_6_)_4_·Dabco: C 37.35, H 3.01, and N 15.68, and the following were found: C 36.88, H 2.92, and N 15.40.

**[Cu_3_(*m*-xpt)_3_(µ-CO_3_)](PF_6_)_4_ ([**3**](PF_6_)_4_) and [Cu_2_(*m*-xpt)_2_(µ-C_2_O_4_)](PF_6_)_2_ ([**4**](PF_6_)_2_):** In a test tube, complex **1a** (100 mg, 0.08 mmol) was dissolved in acetonitrile or DMF (5 mL) and dabco (45 mg, 0.4 mmol) was added. The blue solution turned slightly lighter in the presence of dabco. To the reaction mixture, a small chunk of dry ice was added, and the test tube was enclosed in a CO_2_-purged jar containing diethyl ether for one week. We observed the formation of two distinct types of crystals in the test tube, which were separately analyzed using single-crystal X-ray crystallography. Blue plate-shaped crystals that formed towards the top of the test tube were identified as a carbonate-bridged trinuclear complex, [Cu_3_(*m*-xpt)_3_(µ-CO_3_)](PF_6_)_4_ ([**3**](PF_6_)_4_ (Figure 2). Green needle-like crystals formed towards the bottom of the test tube, which were identified as a co-crystallized complex comprising the carbonate-bridged trinuclear complex [Cu_3_(*m*-xpt)_3_(µ-CO_3_)]^4+^ ([**3**]^4+^) and the oxalate-bridged binuclear complex [Cu_2_(*m*-xpt)_2_(µ-C_2_O_4_)]^2+^ ([**4**]^2+^), with the stoichiometry of [**3**]_2_[**4**](PF_6_)_10_ (Figure 3).

Complex **1b** was also treated in the same mole ratio with dabco, which led to the formation of the same two types of crystals: [**3**](PF_6_)_4_ and [**3**]_2_[**4**](PF_6_)_10_. The proportion of [**3**]_2_[**4**](PF_6_)_10_ in the mixture of products appeared to be higher when **1b** was the reactant (as compared to **1a**). Since **1b** had poor solubility in acetonitrile, we performed further experiments with it in DMF only.

In these experiments, we sometimes observed crystals of the unreacted starting material forming along with [**3**](PF_6_)_4_ and [**3**]_2_[**4**](PF_6_)_10_. The same carbonate and carbonate-oxalate products were obtained in ordinary air (i.e., containing only natural amounts of CO_2_), but in lower yields.

**[**3**](PF_6_)_4_:** FT-IR (cm^−1^): 1458.8 (µ_3_-CO_3_), 823.8, 779.0, and 741.8 (PF_6_). Anal. Calcd for [Cu_3_(*m*-xpt)_3_(µ-CO_3_)](PF_6_)_4_: C 39.96, H 2.70, and N 16.69, and the following were found: C 40.30, H 3.00, and N 16.49. [**3**]_2_[**4**](PF_6_)_10_: FT-IR (cm^−1^): 1659.8 (C=O, oxalate), 1458.8 (C=O, carbonate), 820.0, 777.8, and 743.7 (PF_6_). Anal. Calcd for [Cu_3_(*m*-xpt)_3_(µ-CO_3_)]_2_[Cu_2_(*m*-xpt)_2_(µ-C_2_O_4_)](PF_6_)_10_: C 40.63, H 2.73, and N 16.85, and the following were found: C 39.39, H 3.39, and N 16.94. The discrepancy in microanalysis may have been due to the poorly defined solvents in the crystals. For example, formulae with water of crystallization gave a better agreement with the observed results; however, the solvents in the crystal could not be identified in the X-ray analysis.

**[Cu(*o*-xpt)(PF_6_)]_n_ (5)**: To nitrogen-purged dry DMF (5 mL) in a Schlenk flask, *o*-xpt (25 mg, 0.06 mmol) and [Cu(CH_3_CN)_4_]PF_6_ (24 mg, 0.06 mmol) were simultaneously added. The mixture produced an orange solution, which was stirred for 12 h. Diethyl ether vapor was then slowly diffused into the solution under nitrogen to give **5** (38 mg, 99%) as a bright yellow microcrystalline solid. ^1^H NMR (DMSO-d_6_): 8.71 (br, 4H), 8.30 (br, 4H), 7.87 (br, 8H), 7.62 (s, 8H), 7.31 (br, 4H), and 5.97 (s, 8H). ^13^C NMR (DMSO-*d*_6_): 149.2, 146.8, 145.5, 138.7, 133.6, 132.4, 130.5, 125.3, 124.4, 121.8, and 52.4. NMR showed the presence of DMF as an impurity in the crystalline product. ESI-MS: *m/z* 1059.1542 [Cu_2_(*o*-xpt)_2_(PF_6_)]^+^ (calcd 1059.1537). The product was characterized by single-crystal X-ray analysis as its DMF solvate.

**[Cu_4_(*o*-xpt)_3_(µ_4_-CO_3_)(µ_2_-OH)(µ_2_-CH_3_COO)](PF_6_)_4_ (6):** To DMF (4 mL) in a Schlenk flask, complex **5** (25 mg, 0.02 mmol) was added and stirred under a mixture of CO_2_ and air. The yellow color progressively turned to green. Diethyl ether vapor was then slowly diffused into the reaction mixture, which led to the formation of a precipitate. The solid was separated, redissolved in acetonitrile, and diffused further with diethyl ether vapor to give **6** (18 mg, 85%). IR: 1668 (C=O, acetate) and 1457 (C=O, carbonate). The product was analyzed using single-crystal X-ray crystallography.

**[Cu_2_(*m*-xpt)_2_Br_2_](PF_6_)_2_ (7):** To a stirred solution of **3** (100 mg, 0.05 mmol) in DMF (4 mL), a few drops of 1.0 M BaBr_2_ in DMF were added. The blue solution immediately turned to green, accompanied by the formation of a white precipitate. The precipitate was confirmed to be BaCO_3_: it was separated by centrifugation and dissolved in 1.0 M HCl (2-3 drops), giving the effervescence of carbon dioxide. The green DMF solution was collected and subjected to vapor diffusion with diethyl ether. The solution gave the green blocks of **7** (97 mg, 95%), which was characterized by single-crystal X-ray crystallography. Although the complete refinement was not successful, the analysis was sufficient for the identity of the product (Figure S6). ESI-MS: 1283.033 [Cu_2_(*m*-xpt)_2_Br(PF_6_)_2_]^+^ (calcd 1283.036). Anal. Calcd for [Cu_2_(*m*-xpt)_2_Br_2_](PF_6_)_2_·6H_2_O: C 35.86, H 3.28, N 15.21, and Br 10.84, and the following were found: C 35.65, H 3.47, N 14.70, and Br 9.62.

### 3.3. X-Ray Crystallography

X-ray diffraction data were measured at a low temperature on a Bruker Kappa Apex-II DUO CCD diffractometer (Bruker, Madison, WI, USA) with either MoKα (0.71073 Å) or CuKα (1.54184 Å) radiation. Absorption corrections were performed by a multi-scan technique. The structures were solved using SHELXS [58] and refined using SHELXL [59]. Hydrogen atoms were mostly visible in difference maps and were placed in idealized positions during refinement and treated as riding. Disordered solvent contributions in [**3**](PF_6_)_2_ and [**3**]_2_[**4**](PF_6_)_10_ were removed using the SQUEEZE [60] procedure. The trimeric cation in [**3**](PF_6_)_2_ lay on a two-fold axis, so the carbonate ligand was disordered across that axis. The PF_6_^−^ anion in **5** was rotationally disordered into two orientations. The crystal of **6** was a two-component twin and hydrogen atoms of partially occupied water molecules were not located. The crystal data and refinement parameters of the structures are reported in Table 1 and Table 2; details are available in CIF format as CCDC 2422201–2422204 from https://www.ccdc.cam.ac.uk/structures/.

Two of the structures were of less than ideal quality, as judged by *R* and *wR* factors, and by the esd values for the C–C bond distances. The crystals of [**3**]_2_[**4**](PF_6_)_10_ were small, diffracting relatively weakly, and the asymmetric unit contained a large number of atoms. The crystals of **6** were twinned, with disordered solvent molecules; this limited the quality of the structure that could be obtained. In both cases, repeated crystallization experiments did not yield higher quality crystals.

## 4. Conclusions

This study elucidates the dabco-induced rearrangements in metal–organic supramolecules, emphasizing the distinct roles of dabco and air in facilitating the formation of oxalate and carbonate ions, respectively. The behavior of dabco differs between the macrocycles **1a** and **1b**, despite their subtle differences in ligand environments and coordination geometries. The complex **1a** may accommodate dabco either externally or internally under a nitrogen atmosphere. However, it disassembles and reassembles in the presence of CO_2_ using carbonate ions as a template for the reassembly process. In contrast, complex **1b** participates in a redox reaction with dabco, ultimately oxidizing dabco to oxalate. These reactions demonstrate the influence of the solvent environment: bulk solvents favor carbonate formation possibly due to the presence of water in the solvent, while anhydrous conditions promote oxalate generation. The removal of the carbonate ion from the trinuclear macrocycle [**3**]^4+^ by chemical methods induces a structural rearrangement from a trimer to a dimer.

Further, we investigate the effect of the distance between copper centers by employing *ortho*-xylylenebis(pyridyltriazole), *o*-xpt, as a ligand to coordinate with Cu(I). This modification leads to the formation of a polymeric network rather than a dimeric structure. The resulting polymer exhibits the ability to activate CO_2_ into carbonate while simultaneously facilitating the hydrolysis of acetonitrile to acetate in the presence of air.

These conversions are of interest as facets of metal-based host–guest and redox chemistry. However, they are not directly applicable to large-scale CO_2_ conversion, because they are stoichiometric, not catalytic. Also, the oxalate produced is likely from the oxidation of dabco rather than the reduction of CO_2_.

We are currently exploring the reactivity of structurally similar mononuclear copper complexes of pyridyltriazole or closely related bipyridine derivatives with dabco in the presence of O_2_ and CO_2_.

## Figures and Tables

**Table 1:** Crystal data and structure refinement parameters for compounds [**3**](PF_6_)_4_ and [**3**]_2_[**4**](PF_6_)_10_.

Compound	[**3**](PF_6_)_4_	[**3**]_2_[**4**](PF_6_)_10_
Chemical formula	C_67_H_54_Cu_3_N_24_O_3_(PF_6_)_4_·8CH_3_CN	2[C_67_H_54_Cu_3_N_24_O_3_(PF_6_)_4_]·C_46_H_36_Cu_2_N_16_O_4_(PF_6_)_2_·13.2C_3_H_7_NO
*M*_r_	2342.27	6286.48
Deposition number	2422201	2422202
Crystal system, space group	Monoclinic, *C*2/*c*	Triclinic, *P*$\bar{1}$
Temperature (K)	100.0 (5)	90.0 (5)
*a*, *b*, *c* (Å)	24.980 (2), 16.581 (2), 25.075 (2)	14.0459 (12), 14.8950 (15), 31.686 (3)
α (deg)	90	93.007 (7)
β (deg)	108.560 (8)	101.480 (6)
γ (deg)	90	102.415 (6)
*V* (Å^3^)	9845.9 (17)	6313.1 (10)
*Z*	4	1
Radiation type	Mo Kα	Cu Kα
µ (mm^−1^)	0.82	2.42
Crystal size (mm)	0.13 × 0.11 × 0.09	0.19 × 0.08 × 0.04
*T*_min_, *T*_max_	0.901, 0.930	0.656, 0.909
No. of measured, independent, and observed [*I* > 2σ(*I*)] reflections	34,413, 10,126, 6908	46,744, 21,356, 7056
*R*_int_	0.074	0.109
(sin θ/λ)_max_ (Å^−1^)	0.628	0.588
*R*[*I* > 2σ(*I*)], *wR*(*F*^2^), *S*	0.047, 0.124, 0.98	0.085, 0.238, 0.79
No. of parameters	579	1495
No. of restraints	0	526
∆ρ_max_, ∆ρ_min_ (e Å^−3^)	0.42, −0.42	0.55, −0.42

**Table 2:** Crystal data and structure refinement parameters for compounds **5** and **6**.

Compound	5	6
Chemical formula	C_22_H_18_CuN_8_(PF_6_)·2(C_3_H_7_NO)	C_69_H_58_Cu_4_N_24_O_6_(PF_6_)_4_·0.5C_3_H_7_NO·0.5C_4_H_10_O·CH_3_CN·0.48H_2_O
*M*_r_	749.15	2276.74
Deposition number	2422203	2422204
Crystal system, space group	Monoclinic, *P*2_1_/*c*	Triclinic, *P*$\bar{1}$
Temperature (K)	100.0 (5)	90.0 (5)
*a*, *b*, *c* (Å)	17.7197 (17), 7.9426 (8), 24.116 (3)	19.4804 (10), 20.2673 (10), 22.6368 (11)
α (deg)	90	87.796 (2)
β (deg)	108.252 (5)	88.026 (2)
γ (deg)	90	88.919 (2)
*V* (Å^3^)	3223.4 (6)	8924.1 (8)
*Z*	4	4
Radiation type	Mo Kα	Cu Kα
µ (mm^−1^)	0.81	2.81
Crystal size (mm)	0.42 × 0.16 × 0.07	0.18 × 0.15 × 0.07
*T*_min_, *T*_max_	0.728, 0.946	0.622, 0.827
No. of measured, independent and observed [*I* > 2σ(*I*)] reflections	44,222, 6547, 5476	134,009, 25,452, 16,636
*R*_int_	0.038	0.206
(sin θ/λ)_max_ (Å^−1^)	0.626	0.556
*R*[*I* > 2σ(*I*)], *wR*(*F*^2^), *S*	0.033, 0.085, 1.03	0.089, 0.252, 1.02
No. of parameters	474	2524
No. of restraints	45	27
∆ρ_max_, ∆ρ_min_ (e Å^−3^)	0.34, −0.33	1.31, −0.72

## Data Availability

The data presented in this study are openly available from CCDC at https://www.ccdc.cam.ac.uk/structures/, reference numbers 2422201-2422204.

## Supplementary Materials

The following supporting information can be downloaded at https://www.mdpi.com/article/10.3390/molecules30071430/s1: illustrations of colors of complexes, IR and UV-Vis spectra, and crystal structure drawings.
