# Supplementary material for: Structural Rearrangement in Cyclic Cu(II) Pyridyltriazole Complexes: Oxidation of Dabco to Oxalate and CO2 Conversion to Carbonate
Source: Molecules. 2025 Mar 24;30(7):1430. doi: 10.3390/molecules30071430 (PMC11990479; doi:10.3390/molecules30071430)
Supplement: Supplementary file 1 [file molecules-30-01430-s001.zip › molecules-3527701-supplementary.pdf]

Structural rearrangement in cyclic Cu(II) pyridyltriazole complexes: oxidation of dabco to oxalate and CO_2 conversion to carbonate

Uttam R. Pokharel *, Frank R. Fronczek, and Andrew W. Maverick *

Supporting Information

Table of Contents

Figure S1. Color changes on addition of dabco to solutions of complex **1a** and **1b** S2

Figure S2. UV-Vis spectral changes of $[Cu_2(m\text{-xpt})_2Cl_2](PF_6)_2$ (**1b**) on addition of Dabco. S3

Figure S3. Crystal structure of the protonated dabco salt $[(dabco)_2H^+][dabcoH^+]_2(PF_6^-)_3$ S4

Figure S4. IR spectrum of carbonate complex [**3**]$(PF_6)_4$ S4

Figure S5. IR spectrum of carbonate/oxalate complex [**3**]$_2$[**4**]$(PF_6)_{10}$ S5

Figure S6. Ellipsoid plot of $[Cu_2(m\text{-xpt})_2Br_2](PF_6)_2$. S6

Figure S7. Microcrystalline $[Cu(o\text{-xpt})(PF_6)]_n$, **5** S6

Figure S8. IR spectrum of $[Cu_4(o\text{-xpt})_3(\mu_4\text{-}CO_3)(\mu_2\text{-}OH)(\mu_2\text{-}OCOCH_3)](PF_6)_4$ (**6**) S7

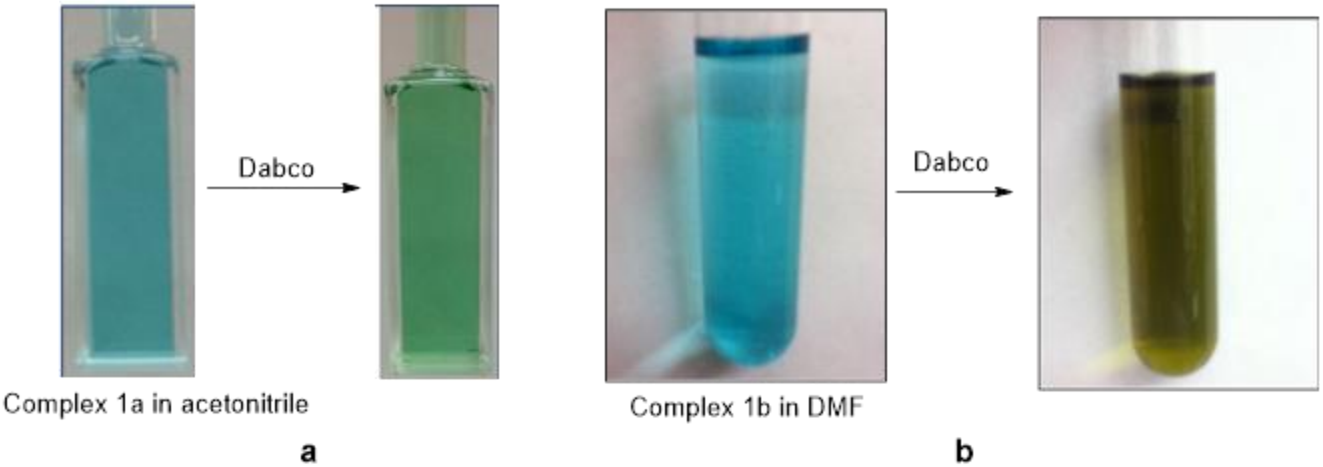

Figure S1. Color changes on addition of dabco: (a) complex **1a** in acetonitrile; (b) complex **1b** in DMF.

Addition of dabco to a solution of **1a** in DMF produces a color change similar to that in CH_3CN. The only combination that produces the dark olive-green color shown in (b) is **1b** + dabco in DMF. We believe this dark color is due to reduction of Cu(II) to Cu(I) by dabco; see the main text and Figure S2 for additional information.

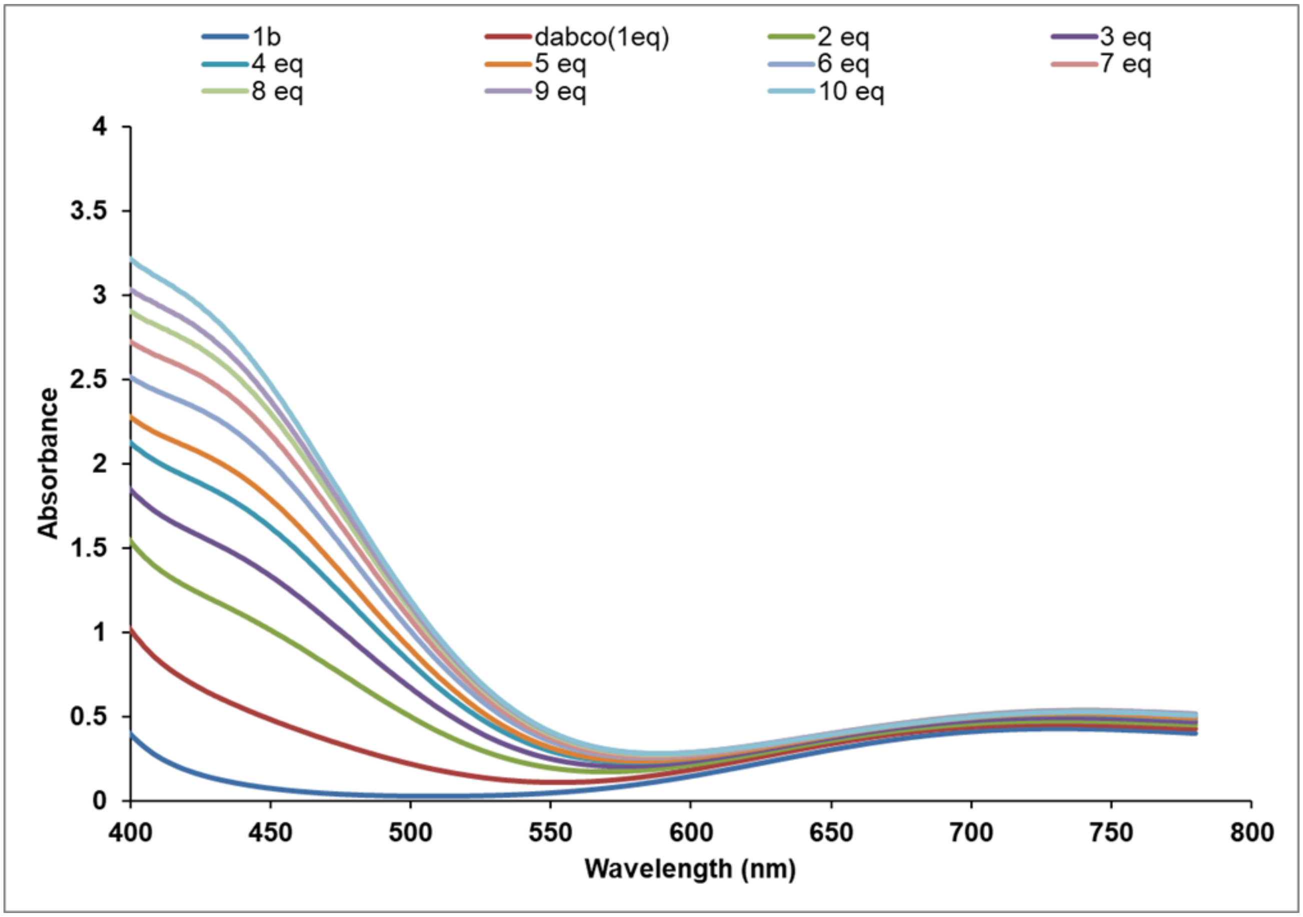

Figure S2. UV-Vis spectral changes of $[Cu_2(m\text{-xpt})_2Cl_2](PF_6)_2$, **1b** (0.74 mM in DMF), on addition of 1-10 eq of Dabco under nitrogen. We assign the intense absorption band at ca. 440 nm to an MLCT transition in the Cu(I) complex that forms when dabco reduces **1b**.

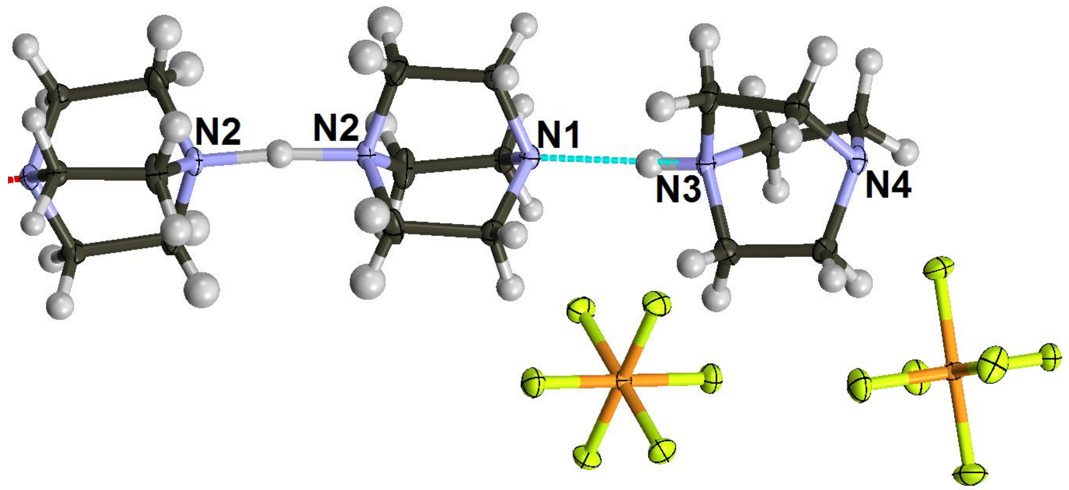

Figure S3. Crystal structure of the protonated dabco salt $[(dabco)_2H^+][dabcoH^+]_2(PF_6^-)_3$ [1]. This salt is one of the products formed when **1a** or **1b** is treated with dabco.

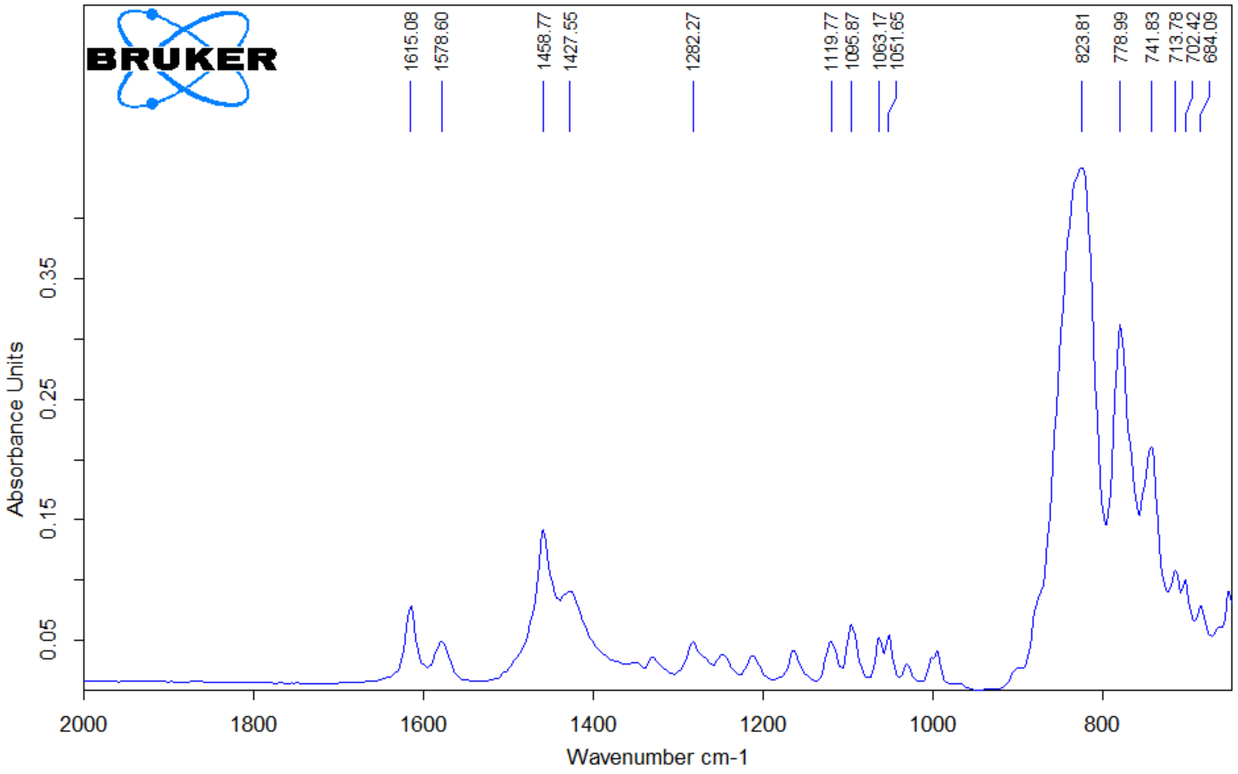

Figure S4. IR spectrum of carbonate complex $[\mathbf{3}](PF_6)_4$

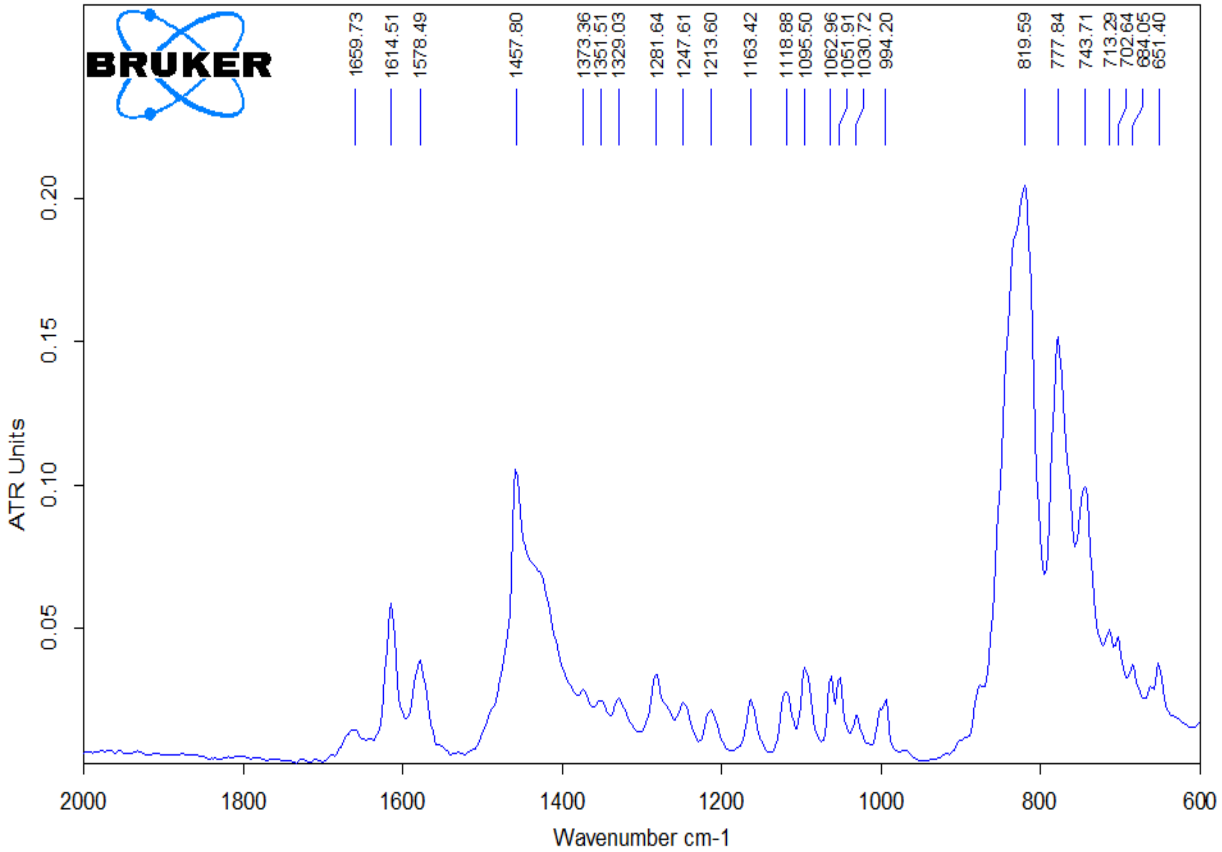

Figure S5. IR spectrum of carbonate/oxalate complex [**3**]$_2$[**4**](PF_6)$_{10}$

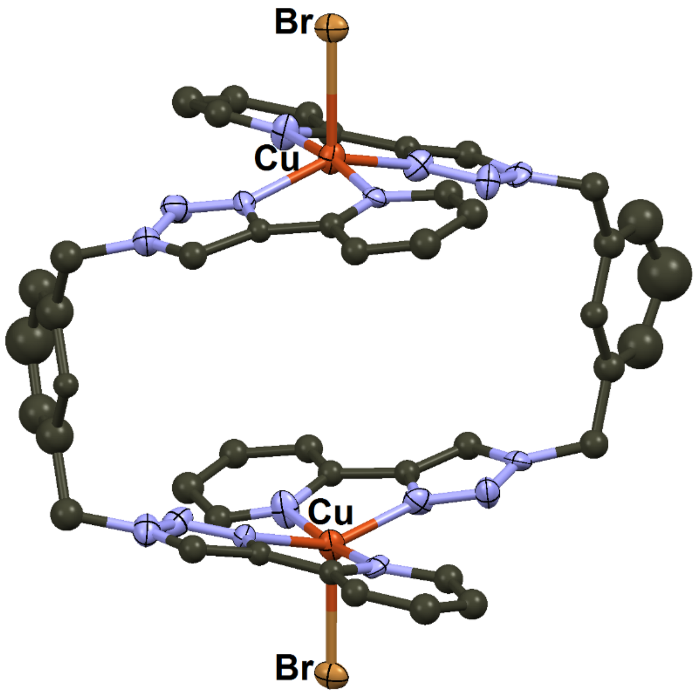

Figure S6. Ellipsoid plot of $[Cu_2(m\text{-xpt})_2Br_2](PF_6)_2$. Cu···Cu = 7.592 Å. Although this compound was clearly identified via single-crystal X-ray analysis, the quality of the crystals was not high enough to support a full refinement.

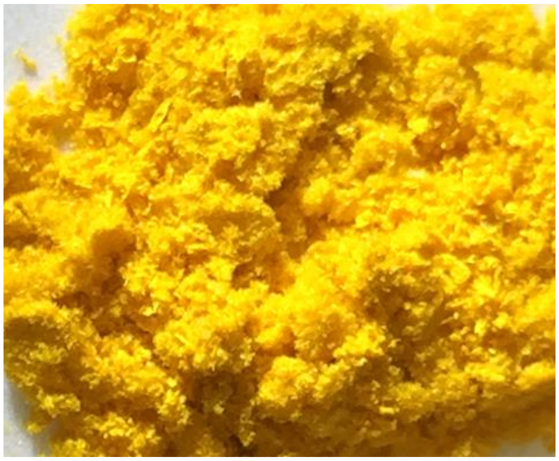

Figure S7. Microcrystalline $[Cu(o\text{-xpt})(PF_6)]_n$, **5**.

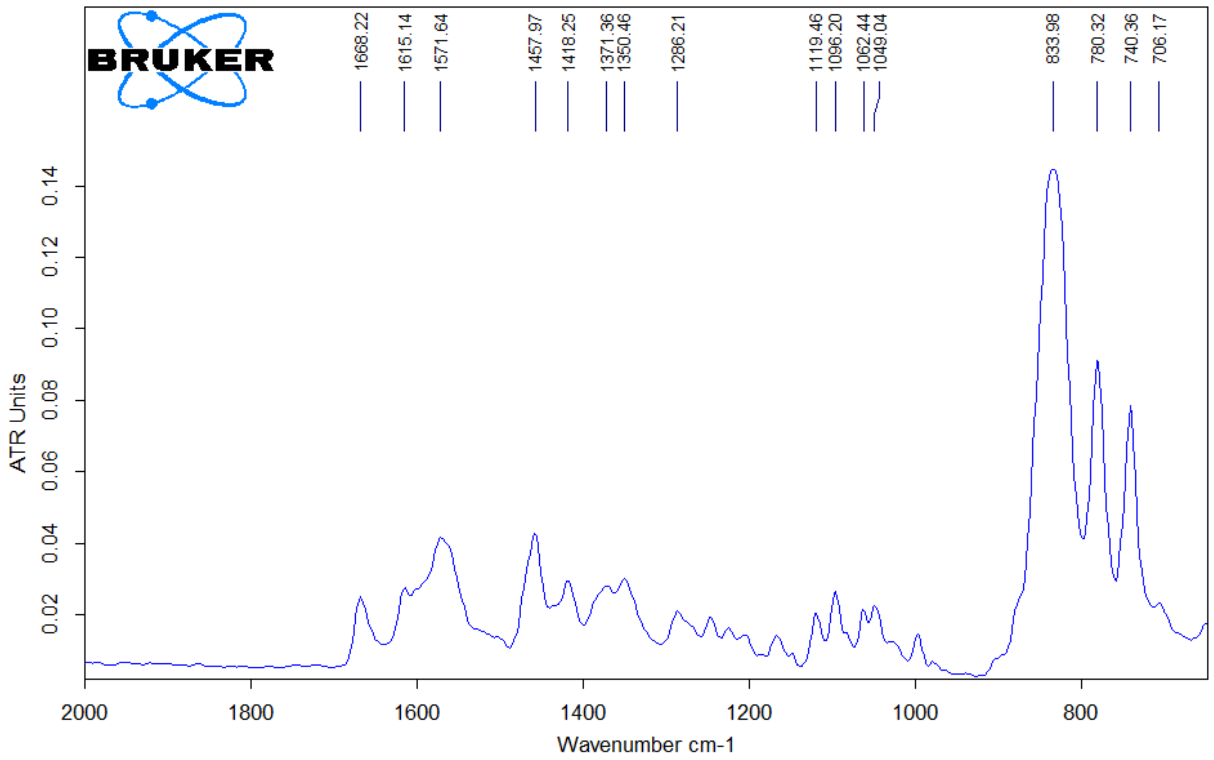

Figure S8. IR spectrum of $[Cu_4(o\text{-xpt})_3(\mu_4\text{-}CO_3)(\mu_2\text{-}OH)(\mu_2\text{-}OCOCH_3)](PF_6)_4$ (**6**).

Reference:

1. Pokharel, U.R.; Maverick, A.W.; Fronczek, F.R. *CSD Commun.* **2014**, doi:10.5517/cc1250bl.
